# Violent and Complex Behaviors and Non-Restorative Sleep Are the Main Features of Disorders of Arousal in Adulthood: Real Picture or a More Severe Phenotype?

**DOI:** 10.3390/jcm12010372

**Published:** 2023-01-03

**Authors:** Greta Mainieri, Giuseppe Loddo, Luca Baldelli, Angelica Montini, Susanna Mondini, Federica Provini

**Affiliations:** 1 Department of Biomedical and NeuroMotor Sciences, University of Bologna, 40139 Bologna, Italy; 2 Department of Primary Care, Azienda AUSL di Bologna, 40100 Bologna, Italy; 3 IRCCS Istituto delle Scienze Neurologiche di Bologna, 40139 Bologna, Italy

**Keywords:** parasomnia, disorder of arousal, slow wave sleep, NREM sleep, sleepwalking, confusional arousal, sleep terror, sleep-related violence

## Abstract

Disorders of arousal (DoA) are NREM parasomnias characterized by motor and emotional behaviors emerging from incomplete arousals from deep sleep. DoA are largely present in pediatric populations, a period during which they are labeled as self-limited manifestations. However, an extensive literature has shown that DoA can persist in adulthood, with different characteristics from childhood DoA. Adult DoA patients usually report excessive daily sleepiness, sleep-related violence during DoA episodes or potentially harmful behaviors, which are rare in childhood. The semeiological features of DoA episodes in adulthood may complicate differential diagnoses with other motor manifestations during sleep, in particular sleep-related hypermotor epilepsy. However, it cannot be excluded that adults with DoA attending sleep centers constitute a more severe phenotype, thus not being representative of adult DoA in the general population. Video-polysomnographic studies of DoA document a spectrum of motor patterns of different complexities, the simplest of which may often go unnoticed. Despite the different complexities of the episodes, neurophysiologic studies showed the co-existence of deep sleep and wakefulness during DoA episodes or even before their onset. These aspects make DoA an ideal model to investigate the mechanisms regulating local sleep, sleep arousal and cognitive functions including spatial and temporal orientation, attention or memory.

## 1. Introduction

**Figure 1:**
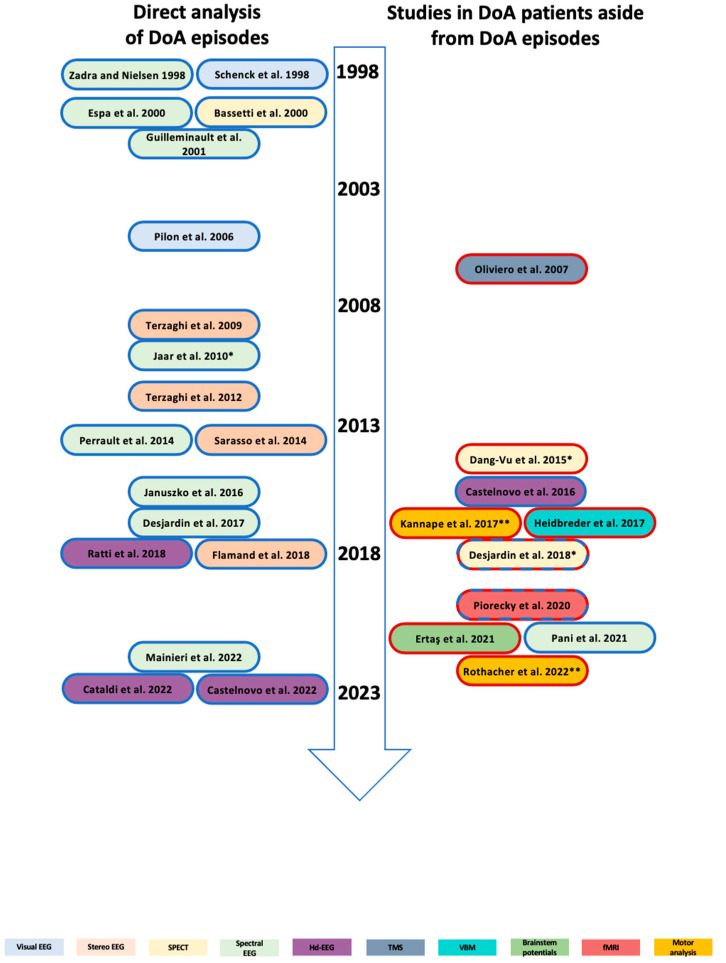
Neurophysiologic studies in DoA. On the left, neurophysiologic studies directly analyzing DoA episodes are illustrated [15,16,17,18,19,20,22,23,24,25,26,30,32,34,36,40,41,42]. On the right are neurophysiologic studies that analyzed DoA patients independently of the episodes (nocturnal sleep detached from episodes/wakefulness) [21,27,29,31,33,35,37,38,43,44]. Listed studies are circled by either blue or red discs, respectively indicating if performed during sleep or wakefulness. “*” and “**”, respectively, indicate studies performed under sleep deprivation or with a specific cognitive load. Studies are illustrated in chronological order. Below: legend of the different neurophysiological methods used in the listed studies, indicated by different colors. Abbreviations: EEG, electroencephalogram; SPECT, single-photon emission computed tomography; Hd, high density; TMS, transcranial magnetic stimulation; VBM, voxel-based morphometry; fMRI, functional magnetic resonance imaging.

Non-rapid eye movement (NREM) sleep parasomnias are characterized by the occurrence of motor and emotional manifestations arising from NREM sleep [1,2,3]. Among them, the group currently referred to as “disorders of arousal” (DoA) is characterized by the occurrence of incomplete awakenings from NREM sleep and particularly from slow wave sleep (SWS). Three main clinical manifestations are currently recognized, namely confusional arousal (CA), sleep terrors (ST) and sleepwalking (SW) [1]. CA, ST and SW may co-occur in the same individual, underpinning that DoA are a spectrum of different clinical manifestations with a similar physiopathologic background. DoA are very frequent during childhood, a period during which they are considered as self-limited, benign manifestations. Traditionally, DoA recognize a genetic predisposition [4,5], over which priming and precipitating factors concur to the occurrence of clinical episodes [6]. This “scheme” has been labeled as the model of the 3Ps [3]. Over the years, both epidemiologic and clinical reports from tertiary adult sleep centers have shown that DoA might persist in adulthood, appear de novo in some cases or even last through elderly life [7]. These aspects have led to the suggestion of a fourth P, i.e., “perpetuating” factors, responsible for the persistence of DoA throughout the life span [8]. Long follow-up studies are lacking for DoA, so several aspects have not been unraveled yet. In particular, it is still unknown if there are differences between DoA arising during childhood and persisting during adult life and DoA emerging de novo in adults or the elderly. The same considerations apply to the long-term consequences of the disorder in relation to diurnal impairment, the presence of daytime sleepiness and reduction in nocturnal sleep quality. Adult patients with DoA search for a sleep center evaluation for several reasons, including physical trauma, violent self- or hetero-directed injuries, diurnal consequences, sleepiness or even forensic implications in some cases. A recent case series showed that episodes of SW may even worsen from childhood to adulthood, pointing out that the term “benign condition” might not be always appropriate for describing adult DoA [9]. In addition, adult DoA might present atypical features [10] or be comorbid with other sleep disorders such as obstructive sleep apnea [11]; hence, a VPSG assessment is often mandatory in order to rule out differential diagnoses or assess the presence of concomitant sleep disorders [12]. However, it is largely unknown whether adults with DoA attending sleep centers are representative of the general population or represent a more severe phenotype. The large use of VPSG, allowing the characterization of DoA semeiology [13,14], has documented a spectrum of motor patterns of increasing complexity, where the simplest episodes, despite being more frequent than the complex ones, may often go unnoticed and not be reported by patients or bed partners [14]. Despite the spectrum of semeiological features, a quite homogeneous neurophysiologic picture is emerging from past or more recent studies through distinct assessments and techniques (Figure 1) [15,16,17,18,19,20,21,22,23,24,25,26,27,28,29,30,31,32,33,34,35,36,37,38,39,40,41,42,43,44], consistent with the idea that diverse states of being such as wakefulness and the different sleep stages may not be mutually exclusive but co-exist and show fluid boundaries in parasomnias [45]. In this paper, we aim to review the principal clinical features of adult DoA, the impact of DoA persistence in adulthood and the related difficulties of reaching a correct diagnosis. Finally, an overview of the neurophysiological background at the basis of DoA is provided.

## 2. Methods

Narrative literature research has been performed on Pubmed and Scopus research databases, with no restriction on articles’ publication date. Published papers up to September 2022 were included. We used the following keywords: “NREM parasomnias” AND (“adults” or “violence”), “disorders of arousal” AND (“adults” or “violence”), NREM parasomnias” AND (“diurnal impairment”), “sleep-related violence”, “confusional arousal” AND (“adults” or “violence”), “sleep terror” AND (“adults” or “violence”) and “sleepwalking” AND (“adults” or “violence”). We considered works only in the English language. Additional papers were inserted from the references of the selected works when relevant.

## 3. Injuries and Violence in Adult DoA Episodes: Which Triggers?

In adults, the presence of a physical injury, potentially harmful behaviors alerting the bed partner or violence during DoA episodes are often one of the principal reasons for consulting a sleep specialist. Described behaviors include moving furniture, running out of bed, jumping from a window, escaping imaginary threats, driving and so on [46]. In rare cases, homicidal or suicidal occurrences have reached forensic attention [47,48,49,50]. Large case series of DoA in adults report injuries in 31–54% of cases [46,51,52,53,54,55,56]. Still, the existence of clinical or external factors promoting more violent episodes in adults is a subject of debate. Among the possible factors, some studies investigated the relationship between the age of onset and the presence of more violent or more frequent episodes, with conflicting results. A recent large study, exploring changes in the frequency and features of DoA episodes during different life periods (childhood, adolescence and adulthood) reported a prevalence of violent SW episodes during adulthood [9]. In addition, the frequency of SW was increased during adulthood, while an opposite trend was observed for ST [9]. It might thus be suggested that a higher frequency of SW episodes during adulthood increases the chance of violent episodes. Other series showed conflicting results, disclosing fewer self-injuries and violent episodes in childhood-onset sleepwalkers in one case [53] and the presence of more violent episodes in patients with early onset (<9 years) of DoA persisting into adulthood than patients with a later onset in another [54]. Another clinical factor often taken into consideration for more violent episodes in DoA is gender. Several studies have reported a prevalence of males among DoA subjects with violent episodes referred to sleep centers [53,54,55,57,58]. This finding is paralleled by similar observations in REM sleep behavior disorder (RBD) where, despite a similar prevalence in both sexes in the general population [59], a male prevalence is recognized in patients attending sleep clinics [60], possibly linked to increased aggressive dream content and more subsequent violent behaviors in men [61]. In DoA, however, previous findings were not confirmed in a recent series, where no difference in gender-related violent episodes was encountered [9]. In this latter study, the authors also included verbal violence in violent behaviors, which could have equalized the two gender groups and lowered the predominance of males that is found when only physical violence is assessed [9].

Among external factors, historically, alcohol consumption has been claimed to trigger SW, especially in some forensic cases, based on the assumption of early studies which showed an increase in SWS after alcohol intake [62,63]. In some series, patients with violent DoA behavior reported more frequently triggering factors such as alcohol ingestion as well as the presence of ST [54]. However, a systematic review on the subject disclosed inconsistent results among published studies, with no direct experimental evidence that alcohol predisposes or triggers SW or related disorders [64]. Other described factors justifying the presence of more violent DoA episodes in adults than in children include the use of sedative–hypnotic drugs [65,66].

External triggers such as noise, touch, physical contact or close proximity to another individual were consistently associated with violent DoA episodes in a review of 32 cases with DoA [67]. In particular, violence was triggered in a different specific manner among the three clinical entities. During CA, a sudden awakening from sleep by someone else could provoke a violent episode. In SW, conversely, victims incidentally encountered the sleepwalker while the episode was already ongoing. In ST, violence seemed to be a reaction to a frightful scene (dreamed or hallucinated) later described by the individual [67]. Finally, internal triggers such as the presence of sleep apnea may also support the presence of more violent DoA episodes in adults than in children [10,68].

## 4. Fatigue, Daytime Sleepiness and Diurnal Impairment

Adult sleepwalkers might also turn to sleep clinics for symptoms such as fatigue, somnolence and diurnal impairment. In a study comparing DoA subjects with normal controls, DoA scored higher on sleepiness and insomnia scales, had higher depressive and anxiety scores and reported a worse quality of life [54]. By means of clinical scales, an association between excessive daytime sleepiness and the presence of SW in adults has been reported in a large epidemiologic US study [69]. Large clinical cohorts disclosed similar proportions of excessive diurnal sleepiness in DoA patients, estimated at around 41–47% [52,54,70,71,72]. A proportion of around 60%, or conversely 15%, was disclosed in other smaller cohorts [53,56]. An objective evaluation of excessive sleepiness through instrumental methods such as multiple sleep latency tests (MSLTs) was performed in a few cohorts [73,74], with initial evidence of a reduced mean sleep latency in 7 out of 10 sleepwalkers in one [73] and no differences in the mean sleep latency in the five MSLT trials between DoA patients and controls in the other [74]. Still, in the study by Lopez et al., patients had reduced sleep latencies for the first two trials. The mechanisms underlying the peak of sleepiness occurring in the early morning in this study remain unclear. The authors suggested a tendency toward delayed sleep phase syndrome, a different response to sleep restriction or a consequence of sleep fragmentation rather than primary daytime sleepiness. With regard to sleep fragmentation, however, the authors did not disclose in the group of patients differences between more “sleepy” and more “alert” phenotypes when assessing slow-wave sleep disruptions with nocturnal sleep recordings [74]. Indeed, no clinical or polygraphic determinants of sleep fragmentation (including SWS arousal, sleep efficiency, clinical DoA episodes, periodic limb movements and sleep apnea) correlated with higher sleepiness in DoA patients [72]. To this extent, another large study on a DoA population, comparing “sleepy” to “non-sleepy” patients, disclosed a specific polygraphic phenotype in patients with higher sleepiness [52]. This consisted of shorter sleep onset latency and a lower number of awakenings in N3 on the first night and higher total sleep time on the second night. Based on these findings, daytime sleepiness in DoA patients appeared not so much related to sleep disruption and parasomnia severity but rather to a specific phenotype indicative of a higher sleep pressure, which also provides the ground for incomplete arousals from SWS [51,52]. Therefore, excessive daytime sleepiness in DoA seems to reflect a higher sleep propensity rather than the consequence of SWS disruption [75]. Of interest, the origin of the diurnal functioning of these patients and its possible relationship with sleepiness is still unknown, and formal cognitive testing has been performed only in a few studies. Two works assessed the performance of a motor procedural task in one case and of verbal memory consolidation in the other in populations of DoA compared to healthy subjects after a normal night of sleep [76,77], disclosing no differences in their performance. Conversely, after sleep deprivation, impairment in inhibitory control, one of the aspects of executive functions, emerged in a group of sleepwalkers [78]. This finding is corroborated by SPECT studies, showing reduced perfusion over frontal regions in wakefulness after sleep deprivation [35]. Based on these and other findings which proved sleep deprivation as a powerful tool to elicit nocturnal episodes [79], it still remains an unresolved question whether a slight diurnal impairment, increased sleepiness or mood alterations are part of the DoA spectrum (as a possible endophenotype) or might represent the consequence of mild chronic sleep deprivation due to SWS fragmentation in some patients.

## 5. Differential Diagnosis in DoA Assessment: The Importance of VPSG

An adult referred to a sleep center for violent episodes during sleep requires an accurate clinical interview, together with a VPSG assessment, which is often mandatory to rule out other diagnoses or demonstrate concomitant sleep disorders. This is especially true when complex nocturnal behaviors are present in the elderly, as recently reported [80], an age group in which the highest suspicion points to RBD. In this case series, four patients (all males) aged > 60 years were referred to the sleep clinics for injurious episodes, disrupted sleep and diurnal impairment, and one of them was affected by Parkinson’s disease [80]. Three out of four patients had a long history of nocturnal episodes, while they started at an older age in one of them, raising a high suspicion of RBD rather than DoA. Even with a long history of nocturnal episodes (suggesting a DoA), the persistence of violent episodes in older people requires a VPSG assessment, necessary to document normal sleep atonia in REM sleep, which excludes a diagnosis of RBD. In addition, in the assessment of adult DoA, some types of sleep-related epilepsy have to be ruled out. In particular, with a history of episodes occurring mostly every night at a high frequency, a differential diagnosis with sleep-related hypermotor epilepsy (SHE) is necessary. SHE is sleep-related focal epilepsy characterized by complex, hypermotor and often bizarre motor behaviors, including asymmetrical tonic or dystonic posture [81]. Notwithstanding, SHE seizures might be also characterized by epileptic wandering, complex automatisms and abrupt awakenings, which could make differential diagnoses with DoA difficult [12,82,83,84]. Some efforts have been made to compare the semeiology of the two conditions [13]. When major episodes are recorded, features such as a waxing and waning temporal pattern, physical or verbal interaction with the environment, modification of the event by external intervention and normal arousal behaviors such as scratching and face rubbing strongly support a diagnosis of parasomnias. Conversely, dystonic-dyskinetic posturing, cycling, rocking or repetitive body movements prevalent in the trunk or legs favor a diagnosis of SHE [13]. All the same, diagnosis may be challenging when only minor episodes are recorded on VPSG. In this direction, a work analyzed and compared minor episodes in SHE, namely paroxysmal arousal (PA) and simple arousal movements (SAMs), the less elaborate episodes in DoA [85]. In these cases, important clues include a more generalized distribution of movements and a shorter duration in PA, while the presence of motor arrests in the motor sequence, behaviors such as exploring the environment and object manipulation were typical of SAMs [85]. Other possible clues derive from sleep staging assessment; traditionally, major events derive from light NREM sleep (stage 1 or 2) in SHE and from stage 3 for DoA [83]. A systematic work analyzing the distribution of major and minor events of both conditions showed that a major episode occurring outside SWS is highly suggestive of SHE, while the presence of minor episodes during stage 3 is supportive of DoA [86].

Recently, a surgical series of patients with cingulate epilepsy described the different semiology of seizures arising from the anterior, middle and cingulate cortex [87]. Interestingly, seizures deriving from an anterior cingulate focus, an area that displays activity similar to wakefulness during parasomnia episodes [24,28], exhibited manifestations quite similar to DoA patients [87]. A systematic comparison of DoA semiology with this specific type of epilepsy could further shed light on DoA mechanisms.

Finally, other factors to consider when assessing an adult DoA include the presence of other concomitant sleep disorders, especially obstructive sleep apnea [11], or the presence of other medical conditions, including psychiatric illness and the use of psychotropic drugs [69]. However, a consistent association with psychiatric illness per se is still unclear, since some psychotropic medications in these cohorts, known to provoke de novo sleepwalking, might have biased this association [69,88]. In summary, in de novo adult patients, a careful assessment of medical and drug history should be performed [89,90,91]. 

According to ICSD-3 [1], the diagnosis of DoA is currently based on clinical criteria, but with the development of new diagnostic instruments, a three-level diagnostic algorithm including a possible, clinical and VPSG-confirmed level may be proposed for DoA assessment [92]. A stepwise algorithm, including a detailed clinical history, questionnaires and homemade videos, provides important clues to nocturnal motor behaviors, but a VPSG is necessary, especially in atypical cases (de novo adult onset, very frequent episodes and episodes in any part of the night) [12,92]. Other diagnostic instruments such as prolonged home recording seem to be a promising approach to increase the probability of capturing typical episodes [93,94].

## 6. Neurophysiologic Basis and Clinical Implications

Beyond scalp EEG, the first study capturing a sleepwalking episode in vivo was a SPECT study, which highlighted activation of the posterior cingulate cortex and anterior cerebellum and diffuse deactivation of fronto-parietal cortices, suggesting a selective activation of thalamo-cingulate arousing systems with persistent quiescence of other thalamo-cortical mechanisms [18]. After this, few stereo-EEG studies recorded by chance in vivo DoA episodes, showing increased activation of motor and limbic cortices, concomitant with the persistence of sleep rhythms/deactivation over fronto-parietal associative cortices and the hippocampus [22,24,25,28]. This general pattern of areas more and less activated has been largely correlated with the classical clinical manifestations of DoA episodes: patients move and might display variable degrees of emotional activation, they seem confused and have an impaired awareness of the environment, they do not feel pain [95] and they display supranormal force and are often amnestic for the event. This general picture might be even more complex since stereo-EEG studies showed inter- and intra-patient variability, as well as a co-existence of both fast and slow EEG rhythms at the same time over the same brain regions [34], hence providing a basis for the different clinical manifestations we can observe during DoA episodes. Recently, important insights were gained from high-density EEG (hd-EEG) studies [36,40,42]. In the work from Cataldi et al. analyzing EEG activity during parasomnia episodes versus normal awakenings in a group of DoA patients, parasomnia episodes arose from a less activated EEG background identified by higher slow wave activity (SWA) and lower beta frequencies in frontal and central brain regions [40]. The authors suggested that, despite a similar spatio–temporal arousal-related slow-wave synchronization process both in parasomnia episodes and normal awakening, parasomnia episodes occur “inappropriately” during periods of high SWA [40]. In other words, it can be advocated that a DoA patient experiences normal awakening when the background sleep is more “activated” and more prone to evolve toward wakefulness, whereas a clinical episode arises when an abnormal persistence of SWA entraps the patient into a state more similar to SWS. Another hd-EEG study on 20 confusional arousal episodes in a single patient highlighted, in a picture of a massive and widespread increase in SWA power over different areas, a relative sparing of the bilateral precuneus and right supramarginal and bilateral angular gyri [42]. The right supramarginal and angular gyri are involved in visuo-spatial attention and redirection of attention towards relevant stimuli, thus suggesting a possible involvement in moving into space during DoA episodes [42]. Indeed, even if traumatic injuries do occur, most episodes pass off without any harm. Precuneus, instead, in addition to its contribution to episodic memory and visuo-spatial processing, is implied in self-reflection and aspects of conscious experience, hinting at a partial regain of consciousness during episodes even if in an altered form [42]. Indeed, episodes of NREM parasomnias are currently known to be underlain by mental content, usually short visual scenes less elaborate than the REM sleep dreaming scenario and characterized by misfortune, high levels of apprehension and self-defense behaviors, with isomorphic features with the motor behavior recorded [70,96,97,98]. With reference to adult DoA, the mental activity associated with episodes is more often recalled than in children [9,96], possibly due to a higher tendency toward proper awakening or at least a more “awake” state related to the progressive decline of SWA with age and the possible co-existence of more wake-like areas than in childhood [96].

Beyond the study of the episode itself, other different neurophysiologic assessments (Figure 1) disclosed increased excitability of motor areas [21,29], decreased activity of frontal areas after sleep deprivation [35], alteration of excitatory brainstem networks [38], a higher ability to perform complex locomotor actions in an unaware condition (under cognitive load) [33] and related EEG markers (higher beta power) corresponding to a higher capacity to perform automatic movements [43]. Even slight structural changes were encountered in an MRI study, characterized by a decrease in gray matter volume of the dorsal posterior cingulate cortex [31]. All in all, this evidence points to subtle differences in DoA patients, even in parts of sleep devoid of clinical episodes as well as normal wakefulness or, in particular, conditions such as sleep deprivation or under certain cognitive loads. Overall, the combination of neurophysiologic evidence makes DoA an ideal model for the study of sleep physiology and sleep arousal. Of note, DoA also provide elements to explore several cognitive domains including spatial and temporal orientation, attention and memory, oneiric content, motor control and the sensory system, embodying an accessible window to the human brain [46].

## 7. Real Picture or More Severe Phenotypes?

Why do some people continue sleepwalking in adulthood while others seem to remit? Which factors contribute to the persistence of DoA episodes? Classically, DoA are intended to be based on a genetic predisposition, on which priming factors influencing the homeostasis of SWS as well as precipitating factors such as apnea, noise or touch produce the episodes [6]. Unresolved priming factors, perpetuating in adulthood, might lead to the persistence of the episodes [8]. Prospective studies with a long follow-up are essential to depict the natural history of NREM parasomnias and the possible factors leading to a better or worse prognosis. In the case of DoA, these data are still lacking due to several reasons. First, DoA are often labeled as benign, self-limiting conditions, where behavioral strategies and safety measures are usually the first approaches for treatment and no approved drug exists to date due to the lack of proper randomized controlled studies [99,100]. Still, as it emerges from adult series, the label of a benign condition might not always be the case [9]. Second, large population studies are lacking partially due to the lack of simple diagnostic instruments with high specificity for the diagnosis of arousal disorders [101] as well as instruments to assess their gravity [71]. Moreover, studies focusing on the analysis of motor patterns in parasomnias have shown that simpler episodes might be the fragment of a more complex one [14], and these episodes might be very frequent when patients are recorded in their home setting [41]. The natural consequence is that we do not know if simpler episodes remain as a disease trait in predisposed individuals who might be completely unaware of them, leaving room for more complex ones only in particular triggering situations. Longitudinal studies, both on a clinical and a polysomnographic basis, are warranted to untangle the factors responsible for the persistence of DoA in adulthood. As an example, stress or stressful events are often reported to worsen or promote the reappearance of DoA episodes. Still, this anecdotal correlation has not been systematically assessed with validated instruments in prospective studies [3]. In this direction, a study demonstrated a prevalence of negative coping strategies in patients with DoA, better exemplified by the subscale consistent with “anxiety rumination” [102]. This result was partly in line with another study showing higher scores on the “anticipatory worry” and “dependence on social attachment” subscales in patients with DoA [103]. All in all, this evidence seems to suggest mechanisms similar to depressive and anxiety disorders, such as difficulty disconnecting from negative thoughts and dysfunctional coping strategies with a tendency for anxiety rumination, which might possibly lead to dysfunction in arousal mechanisms [102], as it already happens in insomnia or mood disorders. In DoA patients in particular, it may be hypothesized that, on a common predisposed and largely genetic background involving SWS homeostasis, some people face a maturational sleep process leading to stable sleep devoid of clinical episodes, while others, in spite of behavioral factors including the tendency to use negative coping strategies in response to stressful situations, maintain a tendency for sleep fragmentation prevalent in SWS, promoting DoA episodes.

## 8. Conclusions and Further Directions

Patients with DoA in adulthood often consult a sleep clinician for reasons other than DoA episodes, including sleep-related harmful behaviors, non-restorative sleep, daytime sleepiness or diurnal impairment. Moreover, adult DoA more often require a VPSG investigation in order to rule out differential diagnoses. Expanding the picture of adult DoA is essential to develop targeted diagnostic and therapeutic strategies, as well as better understand their mechanisms and why they persist in adulthood in some individuals, which are the differences in the variety of clinical manifestations. Most DoA studies are observational; thus, prospective studies are warranted to systematically assess the course of DoA during the lifetime, the related associated features and the possible factors ensuring a better (or worse) prognosis. In addition, sleep clinic studies are probably biased toward patients with a more severe disorder, while patients with milder conditions may be unaware and remain unnoticed. Larger population studies are needed to assess patients with milder phenotypes, patients who eventually remit and still unresolved issues such as the role of gender in NREM parasomnias.

## Data Availability

Not applicable.
